# Untargeted Metabolomics-Based Characterization of the Metabolic Profile and Antioxidant Activity of *Ophiocordyceps sinensis* and Its Substitutes

**DOI:** 10.3390/jof11100740

**Published:** 2025-10-16

**Authors:** Bing Jia, Haoxu Tang, Chuyu Tang, Chao Feng, Yuling Li, Xiuzhang Li

**Affiliations:** State Key Laboratory of Plateau Ecology and Agriculture, Qinghai Academy of Animal and Veterinary Sciences, Qinghai University, Xining 810016, China; bingjia041536@163.com (B.J.); haoxutang0717@163.com (H.T.); chuyutang0410@163.com (C.T.); 18667661285@163.com (C.F.)

**Keywords:** *Ophiocordyceps sinensis*, antioxidant activity, untargeted metabolomics, linoleic acid metabolism

## Abstract

*Ophiocordyceps sinensis* represents a valuable medicinal resource. In this study, mechanisms underlying differences in chemical composition and antioxidant capacity among wild *O. sinensis* (GL), artificially cultivated *O. sinensis* (RG), and product of *O. sinensis* “Bailing” capsules (BL) were systematically investigated via in vitro antioxidant capacity assays and untargeted metabolomics. Results showed GL exhibited the highest total phenol (TPS) content and superior free radical scavenging activity. Additionally, superoxide dismutase (SOD) and peroxidase (POD) activities in RG were higher than those in BL. Correlation analysis of antioxidant indices demonstrated significant positive correlations between total phenols (TPS) and flavonoids (TF) with DPPH radical scavenging, ferric ion reducing antioxidant power (FRAP), hydroxyl radical scavenging rate, and superoxide anion radical scavenging rate (*p* < 0.01). A total of 6729 metabolites were detected, encompassing amino acids and their derivatives, lipids, and nucleotides and their derivatives, among other classes. Furthermore, metabolites exhibited distinct intergroup separation, indicating significant differences in metabolic profiles between *O. sinensis* and its substitute products. KEGG enrichment analysis showed that differential metabolites were mainly enriched in amino acid, lipid, and nucleotide metabolic pathways, among which the linoleic acid metabolic pathway was significantly downregulated. Key metabolites included γ-linolenic acid, 12(13)-EpOME-d, 9-HpODE, etc. Additionally, results of correlation analysis revealed that differential metabolites of lipids, nucleotides, and amino acids exhibited a significant positive correlation with antioxidant indices (*p* < 0.05). These findings suggest that the antioxidant capacity of *O. sinensis* and its substitutes may be regulated via linoleic acid metabolism, providing a theoretical basis for advancing targeted functional development of *O. sinensis* and its substitute products.

## 1. Introduction

*Ophiocordyceps sinensis,* one of the most renowned traditional Chinese medicines in China, offers significant health benefits. This precious traditional Chinese medicine and nourishing fungus has been valued for centuries. *O. sinensis*, also known as ‘Dong Chong Xia Cao’ in China [1], is a unique combination of fungal stroma and deceased larvae that form as a result of *O. sinensis* infecting *Lepidoptera Hepialidae* larvae, utilizing them as the primary source of nutrients [2]. Numerous studies have demonstrated that *O. sinensis* contains a variety of active ingredients including protein, nucleotides, cordycepin acid, ergosterol, and polysaccharides [3,4,5]. Recent pharmacological research has highlighted the antioxidant, immunomodulatory, and anti-fibrotic properties of *O. sinensis* polysaccharides [6,7,8]. Additionally, nucleosides have been found to have diverse effects on macrophages [9]. Owing to the significant medicinal value possessed by *O. sinensis*, its market demand has consistently remained at a high level. This specific parasitic and ecological geographic preference limits the availability of wild resources [10,11,12], leading to a significant rise in the commodity’s price today [13].

The increase in demand and decrease in supply have generated significant interest in the fermentation of *O. sinensis* and the artificial cultivation of *O. sinensis*. Recent years have seen notable advancements in the artificial breeding technology of *O. sinensis* [14], with Sunshine Lake Pharma in Dongguan, China, being a key player. The company has achieved significant technological breakthroughs in the large-scale production of *O. sinensis*, boasting an annual output of over 10 tons [15]. Studies have indicated that artificial *O. sinensis* and wild *O. sinensis* share similar active ingredients [16]. *Hirsutella sinensis* is a stable strain derived from wild *O. sinensis* and characterized as an asexual strain [17,18,19]. Research has demonstrated that *O. sinensis* shares a similar chemical composition (including adenosine, cordycepin acid, sterols, etc.) [14] and pharmacological effects (such as enhanced immunity, anti-fatigue, anti-tumor properties) [20,21] with *O. sinensis*. The fermentation products of *O. sinensis* have been utilized in the production of medical and functional products like capsules, tablets, and granules [22,23,24]. Notably, the Bailing capsule, a health product containing *O. sinensis* powder obtained through liquid deep fermentation, has gained widespread popularity in the market.

In biological systems, the most common free radicals are oxygen-derived free radicals, also known as reactive oxygen species (ROS). ROS encompass several key species, including the superoxide anion radical (O_2_^•−^) and the hydroxyl radical (^•^OH) [25]. The superoxide anion radical is one of the primary ROS generated in biological processes, serving as a precursor for other reactive oxygen species [26,27]. In contrast, the hydroxyl radical is highly reactive and exhibits strong oxidizing properties, capable of inducing severe damage to biological molecules. When ROS levels in the human body surpass a certain threshold and become uncontrolled, they disrupt the balance between the production and removal of pro-oxidant free radicals. This imbalance can lead to damage to various biological macromolecules in the body—the hydroxyl radical, for instance, contributes to lipid peroxidation, damage to membrane proteins, and DNA cross-linking or breakage [28,29]. Consequently, this process accelerates the aging of the body and can contribute to the development of diseases such as cancer, diabetes, rheumatoid arthritis, and Alzheimer’s disease [29]. To counteract the harmful effects of ROS, organisms have evolved a range of defense mechanisms, among which antioxidant enzymes play a pivotal role. Superoxide dismutase (SOD) is ubiquitously found in animals, plants, and microorganisms, serving as the primary defense mechanism against reactive oxygen species—it specifically catalyzes the dismutation of superoxide anion radicals, mitigating their harmful effects. SOD also plays a crucial role in enhancing plant resilience to stress [30,31]. Peroxidase (POD), a type of oxidoreductase, is widely distributed across animals, plants, and microorganisms, with the specific function of eliminating phenolic compounds and peroxides [32]. Catalase (CAT) facilitates the breakdown of hydrogen peroxide into water and oxygen, exhibiting anti-cancer and antioxidant properties [33]. These enzymes, which are referred to as antioxidant enzymes, effectively neutralize excess oxygen free radicals and support essential physiological functions in plants, animals, and humans. Their role in bolstering antioxidant defenses, combating aging, and regulating metabolism is of paramount importance. Research has demonstrated that SOD can play a role in preventing oxidation-related lung diseases [34]. When SOD is used in combination with CAT, it has been shown to decrease the secretion of pro-inflammatory factors, effectively suppressing the inflammatory response, and safeguarding cells from oxidative damage caused by ROS [35]. Consequently, enhancing the body’s antioxidant capacity by consuming exogenous antioxidants has emerged as a viable approach to alleviating oxidative stress and mitigating the risk of various diseases [36].

Natural antioxidants are generally non-toxic and rarely cause adverse side effects. As a result, there is growing interest in discovering antioxidants from natural sources such as plants, bacteria, and fungi, particularly phenolic derivatives, peptides/protein hydrolysates, phospholipids, and polysaccharides. The potential applications of these compounds in the food industry and preventive medicine are being explored [37]. Numerous studies have shown that fermented *O. sinensis* and artificial *O. sinensis* can be viable alternatives to wild *O. sinensis* in terms of efficacy [38]. With the development of multi-omics techniques, metabolomics is a discipline after genomics and proteomics, which provides the best and most intuitive response to dynamic biological changes in specific processes [39,40]. Edible mushrooms are rich in polysaccharides, sterols, lipids, proteins, and other metabolites, which have extensive scientific value [41,42]. The purpose of this study was to compare the active ingredients associated with antioxidant activity in wild *O. sinensis*, artificial *O. sinensis* and fermented *O. sinensis* ‘Bailing’ capsules, and to analyze the metabolic profiles of *O. sinensis* and its subsistence by non-targeted metabolomics methods. In addition, the relationship between differential metabolites and antioxidant activity of *O. sinensis* and its substitutes was explored through correlation analysis, which provided basic data for the further development and utilization of *O. sinensis* and its substitutes.

## 2. Materials and Methods

### 2.1. Fungal Sample Materials

**Figure 1:**
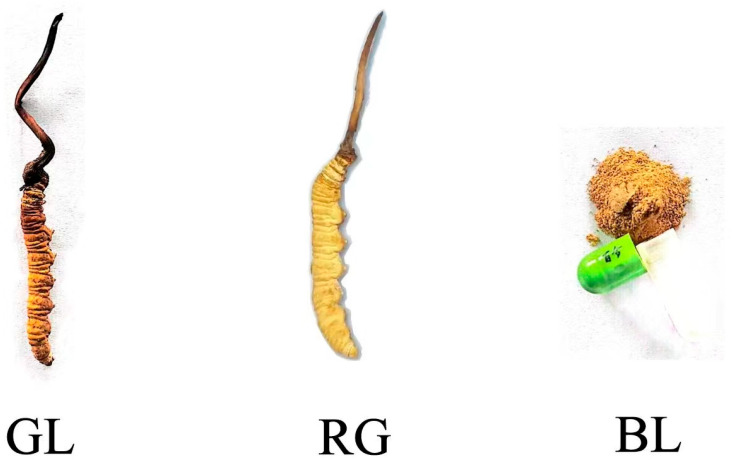
*Ophiocordyceps sinensis* and its substitutes. (GL) Wild *O. sinensis*; (RG) artificially cultivated *O. sinensis*; (BL) fermented *O. sinensis* ‘Bailing’ capsules.

Wild *O. sinensis* was purchased from Baihuitang Biotechnology Co., Ltd. located in Xining City, Qinghai Province, China. Artificially cultivated *O. sinensis* was obtained from Guangdong Dongguang Pharmaceutical Co., Ltd., (Dongguan, China) while fermented *O. sinensis* ‘Bai ling’ capsules were purchased from a local pharmacy. Each of the three samples obtained was in a dry form. Detailed information regarding the samples can be found in Figure 1.

### 2.2. In Vitro Antioxidant Assay

SOD kit, POD kit, CAT kit, DPPH kit, FRAP kit, hydroxyl radical kit, total phenol kit, and flavonoid kit were sourced from Suzhou Keming Biotechnology Co., Ltd. (Suzhou, China). The superoxide anion reagent kit and BCA protein kit were obtained from Beijing Solarbio Technology Co., Ltd. (Beijing, China). All experimental procedures were meticulously conducted following the guidelines provided. Specific information regarding each assay method can be found in Table 1.

### 2.3. Untargeted Metabolomics Profiling

#### 2.3.1. QC Sample Preparation and Sample Extraction

A 5424 R cryogenic high-speed centrifuge (Eppendorf Hamburg, Germany) and a KQ-250 DE ultrasound clarifier (Kunshan, China) were used for the study. Formic acid (chromatographically pure, LC-MS grade) from Shanghai Aladdin Biochemical Technology Co., Ltd., acetonitrile (chromatographically pure, LC-MS grade) and methanol (chromatographically pure, LC-MS grade) from Merck (Darmstadt, Germany).

Wild *O. sinensis*, artificially cultivated *O. sinensis*, and fermented *O. sinensis* ‘Bailing’ capsules were first placed in a freezer dryer (Scientz-100 F is from Ningbo Xinzhi Biotechnology Co., Ltd., Ningbo, China.) for vacuum freeze-drying for 63 h; the dried samples were then ground into powder using a grinder (MM 400, RETSCH, Haan, Germany) at 30 Hz for 1.5 min. An electronic balance (MS105 DM) (Zurich, Switzerland) was used to weigh 50 mg of the sample powder, and 1200 μL of −20 °C pre-cooled 70% methanol aqueous solution containing the internal standard was added (if the sample weight was less than 50 mg, the extractant was added at the ratio of 1200 μL per 50 mg sample); the internal standard extraction solution was prepared by dissolving 1 mg of standard in 1 mL of 70% methanol aqueous solution to prepare a 1000 μg/mL standard stock solution, and the 1000 μg/mL stock solution was further diluted with 70% methanol aqueous solution to prepare a 250 μg/mL internal standard solution. The mixture was vortexed every 30 min, with each vortex lasting 30 s, and this vortexing process was repeated a total of 6 times. After centrifugation at 12,000 rpm for 3 min, the supernatant was aspirated, and the sample was filtered through a microporous membrane (0.22 μm pore size) and then stored in an injection flask for subsequent UPLC-MS/MS analysis.

#### 2.3.2. LC-MS/MS Analysis

Samples were separated by LC-30A Ultra-High Performance Liquid Chromatograph (Shimadzu, Kyoto, Japan) Waters ACQUITY UPLC HSS T3 Column (1.8 μm, 2.1 mm × 100 mm). The instrument column temperature was maintained at 40 °C, with a flow rate of 0.40 mL/min and an injection volume of 4 μL. The mobile phase consisted of phase A (ultrapure water containing 0.1% formic acid) and phase B (acetonitrile containing 0.1% formic acid). Throughout the analytical process, samples were stored in the autosampler at 4 °C. To eliminate the effect of fluctuations in the detection signal of the instrument, the samples are analyzed continuously in random order. The gradient separation process is detailed in Table 2.

#### 2.3.3. Mass Spectrometry Conditions

Primary and secondary spectra of samples were collected using the AB Triple TOF 6600 mass spectrometer (Shanghai Applied Protein Technology Co., Ltd., Shanghai, China). The source conditions in both ESI^+^ and ESI^−^ mode after separation were as follows: the acquisition time was 10 min; The ionization voltages are 5000 V and −4000 V, respectively; The temperature of the ion source is 550 °C; Spray gas (Ion Source Gas 1) is 50 psi; Auxiliary heating gas (Ion Source Gas 2) is 60 psi; Curtain gas is 35 psi; ESI^+^ is 60 V in Declustering Potential, ESI^−^ is −60 V; The collision energies of MS1 are ESI^+^ and ESI^−^ 10 V respectively; MS 2 collision energy ESI^+^ is 30 V, ESI^−^ Yes −30 V; The collision energy step size is 15 V.

#### 2.3.4. Data Processing

All quantitative data for the determination of in vitro antioxidant capacity followed a normal distribution, and data compilation and statistical processing were performed using Microsoft Excel 2020. Significance testing of data differences and analysis of variance were conducted via IBM SPSS Statistics 26.0 software, with the significance level set at *p* < 0.05. For untargeted metabolomics data, raw MS data were first converted to mzML format using Proteo Wizard, followed by preprocessing via XCMS workflows (peak extraction, alignment, retention time correction). Peak picking was done with the cent Wave algorithm. Peak grouping and annotation of isotopes/adducts were conducted via CAMERA. Data screening included filtering out peaks with >50% missing rate per group and imputing missing values by KNN. Peak area correction was performed using SVR. Corrected/filtered peaks were identified via multi-dimensional database searches (in-house, public, prediction libraries; met DNA algorithm). Final screening criteria included a composite identification score ≥ 0.5 and a CV < 0.5 for QC samples. Positive/negative ion mode datasets were merged, with metabolites retained based on the highest qualitative confidence and smallest CV. Multivariate statistical analysis first employed principal component analysis (PCA) to evaluate the stability of the entire analytical workflow, followed by orthogonal partial least squares-discriminant analysis (OPLS-DA) to distinguish overall differences in metabolic profiles between groups and screen for differentially accumulated metabolites (DAM). DAM were identified based on the variable importance in projection (VIP) values from the OPLS model and *p*-values, with those meeting VIP > 1 and *p* < 0.05 defined as DAMs [43,44]. The generation and analysis of volcano plots, cluster heatmaps and Venn diagrams were performed using a cloud platform (https://cloud.metware.cn/, accessed 10 July 2025). Functional annotation of DAMs and metabolic pathway analysis were conducted through compound annotation based on information from the Kyoto Encyclopedia of Genes and Genomes (KEGG, https://www.genome.jp/kegg/, accessed 15 July 2024). Correlation analysis was carried out using the ChiPlot tool (https://www.chiplot.online/, accessed 25 July 2025).

## 3. Analysis of Results

### 3.1. Comparative Analysis of In Vitro Antioxidant Activity

**Figure 2:**
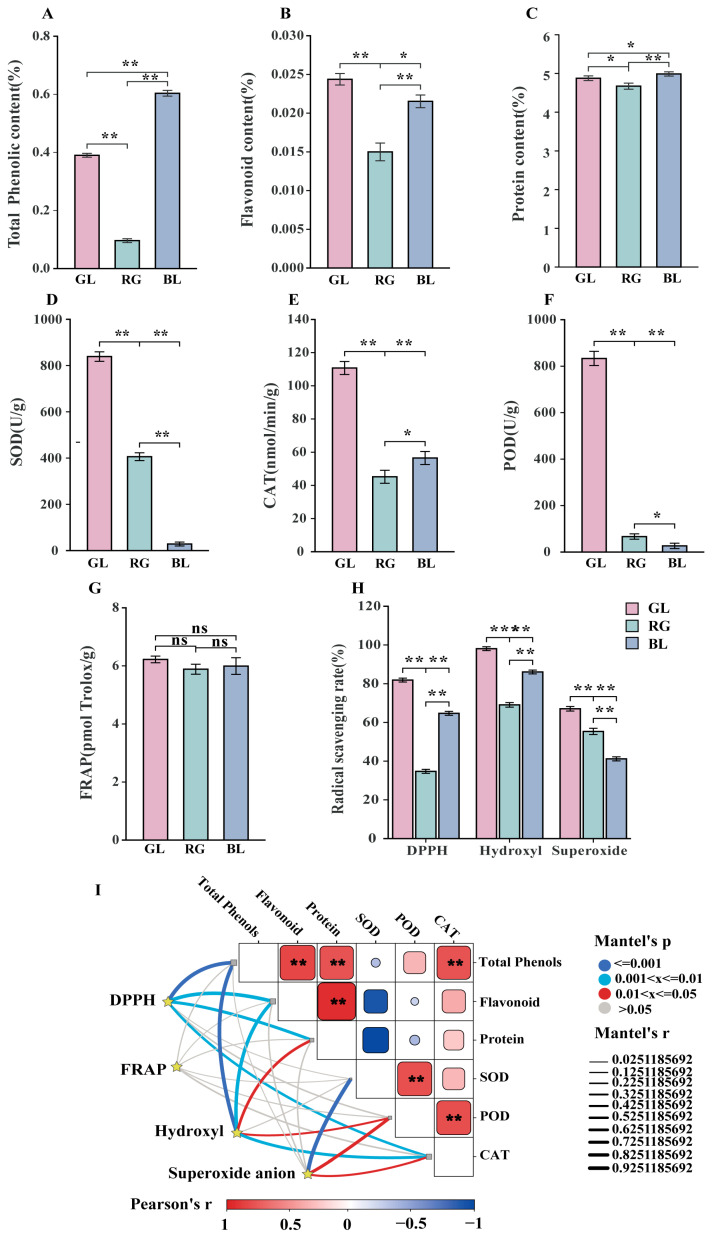
Compares the antioxidant indices of *O sinensis* and its substitutes. (**A**) Total phenolic content; (**B**) Total flavonoid content; (**C**) Protein content; (**D**) Superoxide dismutase (SOD); (**E**) Catalase (CAT); (**F**) Peroxidase (POD); (**G**) Ferric ion reducing antioxidant power (FRAP); (**H**) 2,2-diphenyl-1-picrylhydrazyl radical scavenging capacity (DPPH·); Hydroxyl radical scavenging capacity (^•^OH); Superoxide anion radical scavenging capacity (O_2_^•−^); In figures (**A**–**H**), the error bars represent the mean ± standard deviation (SD) of each group of data, reflecting the degree of data dispersion and the reliability of the mean. (**I**) Interactive Mantel test-based correlation heatmap analysis of DPPH, FRAP, ^•^OH, O_2_^•−^·, enzymatic substances, and active substances. The upper part of the analysis presents the correlation heatmap between enzymatic antioxidant substances and active substances. The lower part shows the Mantel test analysis. The color scheme in the heatmap represents the correlation coefficients among active substances, where red indicates a positive correlation, blue indicates a negative correlation, and gray indicates a weak correlation. In addition, the thickness of lines in the network reflects the strength of Mantel correlations; thicker lines indicate a stronger relationship between antioxidant substances and the four antioxidant indices. “**” indicates extremely significant differences (*p* < 0.001), “*” indicates significant differences (*p* < 0.05), and “ns” indicates no significant differences (*p* > 0.05).

Through the determination of relevant antioxidant indicators, the differences in antioxidant capacities between *O.sinensis* and its substitutes were analyzed. The protein contents of GL, BL and RG were 4.9 ± 0%, 5.0 ± 0% and 4.7 ± 1%, respectively (Figure 2C). When comparing the total phenolic content, GL had the highest values of 0.024 ± 0.001%, which was similar to BL 0.022 ± 0.001% (Figure 2A); In terms of flavonoid content, GL, RG and BL were 0.390 ± 0.005%, 0.604± 0.006% and 0.096 ± 0.004%, respectively (Figure 2B), and the three comparison groups had extremely significant differences (*p* < 0.01). The SOD activities of GL, RL and BL were 838.88 ± 20.71 μ/g, 28.32 ± 5.55 μ/g and 405.84 ± 16.79 μ/g (Figure 2D). In terms of CAT activity, GL, RG and BL recorded 110.70 ± 3.91 nmol/min/g, 56.50 ± 3.54 nmol/min/g, and 45.20 ± 3.12 nmol/min/g, respectively (Figure 2E). Finally, In terms of POD activity, GL, RG and BL showed 833.33 ± 30.55 μ/g, 26.67 ± 11.55 μ/g, and 66.67 ± 11.54 μ/g, respectively (Figure 2F). It is worth noting that GL showed the highest content among SOD, POD, and CAT. The antioxidant activity showed that the contents of FRAP in GL, RG and BL were 6.22 ± 0.11 pmol Trolox/g, 5.99 ± 0.29 pmol Trolox/g and 5.89 ± 0.17 pmol Trolox/g, respectively, with no significant difference (*p* > 0.05) (Figure 2G). The ^•^OH scavenging rate of the three samples was above 50%, except for the O_2_^•−^ scavenging rate of RG which was significantly higher than that of BL (*p* < 0.01), and the highest content of DPPH free radical scavenging rate and ^•^OH scavenging rate was GL, followed by RG (Figure 2H). In addition, correlation analysis was carried out to explore the relationship between active substances, enzyme antioxidants and antioxidant activity. The Mantel test results showed a significant positive correlation between protein and ^•^OH clearance and DPPH clearance (Figure 2I). CAT activity, total phenolic content, and flavonoid content were significantly positively correlated with ^•^OH clearance and DPPH clearance. SOD and POD were significantly positively correlated with O_2_^•−^, with correlation coefficients of 0.974 and 0.915, respectively (Figure 2I). It is shown that these active substances affect the antioxidant activity of ^•^OH, O_2_^•−^ and DPPH. In summary, total phenols, proteins, flavonoids, and CAT mainly affect the antioxidant activity of ^•^OH and DPPH, while these active substances have no significant effect on FRAP’s antioxidant activity. These substances play a crucial role in the antioxidant activity of *O. sinensis* and its substitutes in the ^•^OH, O_2_^•−^, and DPPH antioxidant activity.

### 3.2. Analysis of Metabolite Profiles

The study employed LC-MS/MS technology for untargeted metabolomics to generate total ion chromatograms (TICs) for QC, GL, BL, and RG. The results showed that the total ion flow of metabolites had a high overlap. The retention time and peak intensity were consistent, indicating that the MS maintained a stable signal in positive mode (Figure S1) and negative mode (Figure S2) when mass spectrometry detected the same sample at different times. The high stability of the instrument provides an important guarantee for the repeatability and reliability of the data.

**Figure 3:**
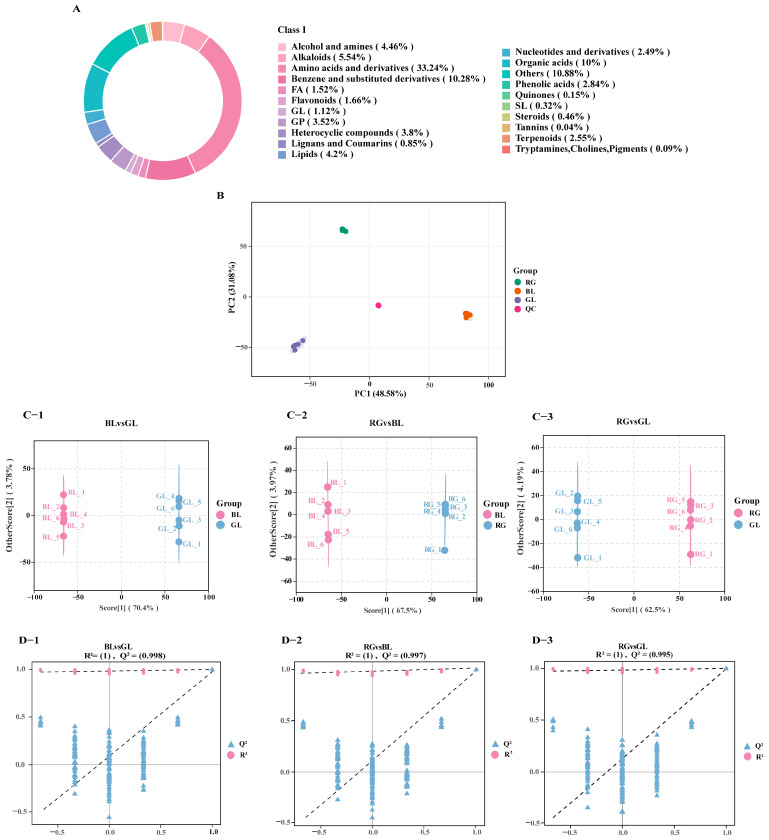
Metabolites detected in GL, RG, and BL, along with multivariate statistical analysis of these metabolites, are presented here. (**A**) Metabolic classification of GL, RG, and BL at the superclass level. (**B**) PCA of RG, BL, GL, and QC samples. (**C-1**–**C-3**) OPLS-DA score plots for BL vs. GL, RG vs. BL, and RG vs. GL, respectively. (**D-1**–**D-3**) OPLS-DA permutation tests corresponding to BL vs. GL, RG vs. BL, and RG vs. GL, the two dashed lines represent the regression line of (R^2^Y) and the regression line of (Q^2^) respectively. They are used to show the trend of (R^2^Y) and (Q^2^) changing with the permutation retention during the permutation testing process, helping to judge the reliability of the OPLS-DA model and so on.

Metabolites in GL, BL, and RG were analyzed using LC-MS/MS technology integrated with public databases. A total of 6792 known metabolites were detected across these samples, comprising 4048 ESI^+^ metabolites and 2744 ESI^−^ metabolites(S1). These identified metabolites were categorized into 21 superclasses, such as amino acids and their derivatives, benzene and its derivatives, organic acids, alkaloids, lipids, glycerophospholipids, flavonoids, heterocyclic compounds, aliphatic acyls, nucleotides and their derivatives, and terpenes (Figure 3A). Notably, amino acids and their derivatives accounted for the largest proportion of metabolites, at 33.24% (2258 species), followed by other metabolites at 10.88% (739 species), benzene and its derivatives at 10.28% (698 species), organic acids at 10% (679 species), and alkaloids at 5.5% (376 species).

Principal component analysis (PCA) was performed to analyze metabolite profiles of GL, BL, RG, and quality control (QC) samples. This analysis uncovered distinct inter-group differences and intra-group variability among the sample groups. The PCA score plot demonstrates that principal component 1 (PC1) explains 48.5% of the total variance, with principal component 2 (PC2) accounting for 31.08%. Obvious inter-group separation was observed among samples, indicating significant differences in metabolite compositions between GL, BL, and RG. Notably, GL exhibited significant intra-group variability. QC samples clustered tightly near the center of the PCA plot, indicating minimal measurement bias and thereby validating the robustness of the analytical method and the reliability of metabolomic data across all samples (Figure 3B). In contrast to the unsupervised PCA model, orthogonal partial least squares-discriminant analysis (OPLS-DA) is a supervised discriminant statistical method with superior classification and predictive performance. For optimized inter-group separation, OPLS-DA was applied for further analysis following exclusion of QC samples. In OPLS-DA models, R^2^X and R^2^Y denote the explanatory power of the model for the X (predictor) and Y (response) matrices, respectively, while Q^2^ reflects the predictive ability of the model. OPLS-DA models were constructed to compare metabolite compositions between BL vs. GL (R^2^X = 0.714, R^2^Y = 1, Q^2^ = 0.998; Figure 3D-1), RG vs. BL (R^2^X = 0.714, R^2^Y = 1, Q^2^ = 0.997; Figure 3D-2), and RG vs. GL (R^2^X = 0.667, R^2^Y = 1, Q^2^ = 0.995; Figure 3D-3). All pairwise comparisons yielded high R^2^X, R^2^Y, and Q^2^ values. To avoid overfitting of the supervised OPLS-DA models during construction, permutation tests were conducted to validate model validity. Gradual decreases in R^2^ and Q^2^ values of randomized models indicated the absence of overfitting in the original models (Figure 3D-1–D-3), confirming that inter-group metabolite separation was statistically significant.

### 3.3. Screening of Differentially Accumulated Metabolites (DAM)

**Figure 4:**
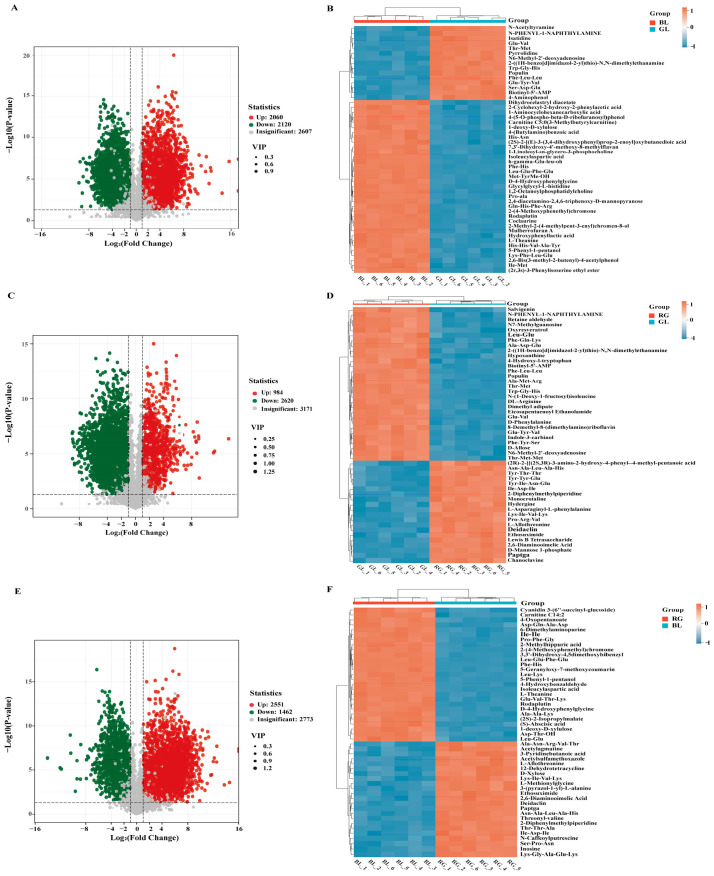
Differential metabolite analysis of GL, RG, and BL. (**A**) Volcano plot of RG vs. BL; (**B**) Hierarchical clustering of BL vs. GL; (**C**) Volcano plot of BL vs. GL; (**D**) Hierarchical clustering of RG vs. BL; (**E**) Volcano plot of RG vs. GL; (**F**) Hierarchical clustering of RG vs. GL.

Significant differential accumulated metabolites (DAM) across pairwise comparisons of *O. sinensis* samples were filtered using the criteria of *p*-value < 0.05 and VIP > 1, with results visualized through volcano plots (Figure 4A–E) and Venn diagrams (Figure 4A). To visually characterize DAM abundance patterns among GL, RG, and BL, all significant DAMs were subjected to hierarchical clustering analysis, focusing on the top 50 metabolites ranked by VIP values (Figure 4B–F). A total of 4013 DAMs were identified in the RG vs. BL, with 2551 DAMs up-regulated and 1462 down-regulated (Figure 4A). In the BL vs. GL comparison, 4180 DAMs were detected, including 2060 up-regulated and 2120 down-regulated compounds (Figure 4C). For the RG vs. GL, 3604 DAMs were identified, consisting of 984 up-regulated and 2620 down-regulated metabolites (Figure 4E). These DAMs were primarily classified into functional categories such as amino acids and their derivatives, heterocyclic compounds, benzene derivatives, nucleotides and their derivatives, organic acids, and alkaloids. Notably, 1207 overlapping DAMs were identified across comparisons, among which the overlapping DAMs in each comparison group exhibit distinct relative abundances—for example, 9,12-Octadecadiynoic acid shows a relatively high content in the wild comparison group, while 9-Octadecynoic acid has a relatively high content in the artificial comparison group—serving as key markers for distinguishing between wild and artificially cultivated *O. sinensis*. This observation indicates distinct variations in nutrient composition between wild and artificially cultivated *O. sinensis*.

### 3.4. KEGG Enrichment Pathway Analysis

**Figure 5:**
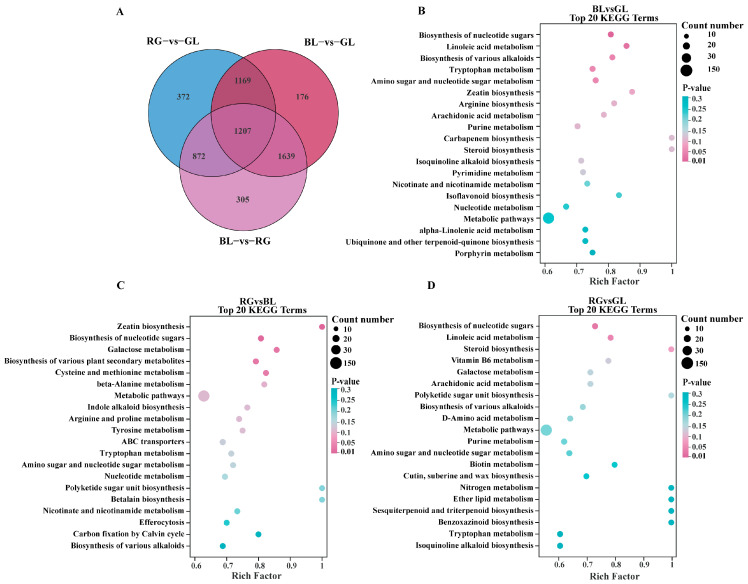
Venn diagram and pathway analysis of differential metabolites. (**A**) The Venn diagram shows overlapping and unique metabolites among groups. (**B**–**D**) KEGG enrichment analyses of differential metabolites between groups. The abscissa represents the enrichment ratio, and the ordinate displays the top 20 pathway terms. Larger bubbles indicate pathways containing more differential metabolites; the bubble color transitions from blue to pink, corresponding to decreasing enrichment *p*-values (i.e., more significant enrichment).

**Figure 6:**
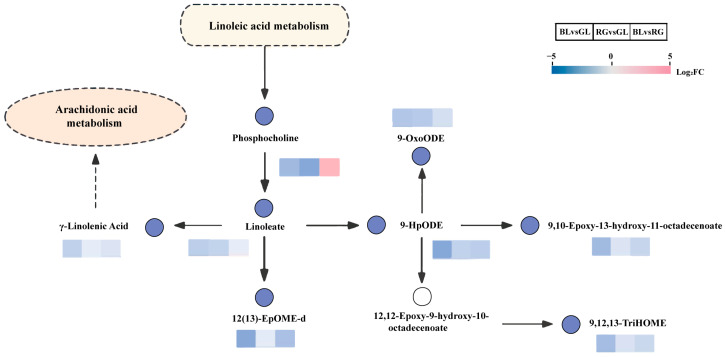
Overview of metabolic pathways mapping key metabolites potentially regulated in pairwise comparisons of BL vs. GL, RG vs. GL, and RG vs. BL. Colored boxes for each metabolite indicate the corresponding log_2_ fold change (log_2_FC) values. Small red rectangles indicate significant up-regulation of metabolites between groups, while small blue rectangles indicate significant down-regulation of metabolites between groups. Small white rectangles indicate non-significant differences between groups. Solid arrows represent facilitative relationships, and dotted boxes denote distinct metabolic pathways.

The Kyoto Encyclopedia of Genes and Genomes (KEGG) database (https://www.genome.jp/kegg/, accessed on 10 July 2025) serves as a core resource for elucidating the mechanisms underlying metabolic pathway perturbations across sample groups through enrichment analysis of DAM. In this study, the top 20 most significantly enriched metabolic pathways were prioritized for further investigation, with differential metabolites in each comparison group annotated and mapped to their respective pathways. Across the three pairwise comparisons, differential metabolites in BL vs. GL, RG vs. BL, and RG vs. GL were assigned to 105, 97, and 97 pathways, respectively, with key pathways visualized using bubble plots (Figure 5B–D). Notably, nucleoside biosynthesis emerged as a significantly upregulated pathway across all three comparison groups. Specifically, in the BL vs. GL comparison, nucleoside biosynthesis, linoleic acid metabolism, and the biosynthesis of various alkaloids ranked as the top three pathways with significant differences (Figure 5B). For the RG vs. BL comparison, the leading pathways with significant differences were zein biosynthesis, nucleotide biosynthesis, and galactose metabolism (Figure 5C). In the RG vs. GL comparison, three pathways exhibiting marked differences included nucleotide sugar biosynthesis, linoleic acid metabolism, and steroid biosynthesis (Figure 5D). These findings suggest that BL, RG, and GL likely possess distinct metabolite profiles, which may be linked to the antioxidant capacity of *O. sinensis*. Furthermore, based on KEGG annotations and enrichment plots, the linoleic acid metabolic pathway was selected for in-depth analysis to characterize metabolic regulatory changes (Figure 6). Within this pathway, metabolites including γ-linolenic acid, linoleic acid, and lecithin were downregulated; such downregulation of these differential metabolites may be strongly associated with antioxidant activity or enzyme activity.

### 3.5. Correlation Analysis

**Figure 7:**
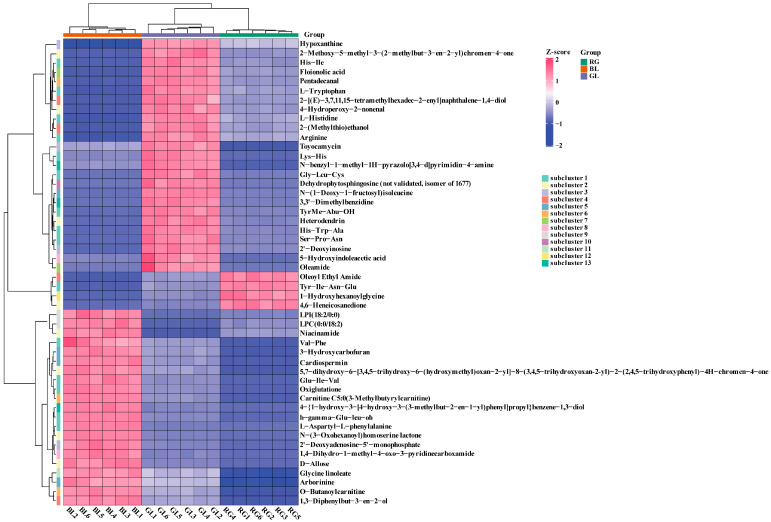
Hierarchical cluster analysis (HCA) of the top 50 metabolites among GL, BL, and RG. Red indicates higher metabolite abundance, while blue indicates lower metabolite abundance.

**Figure 8:**
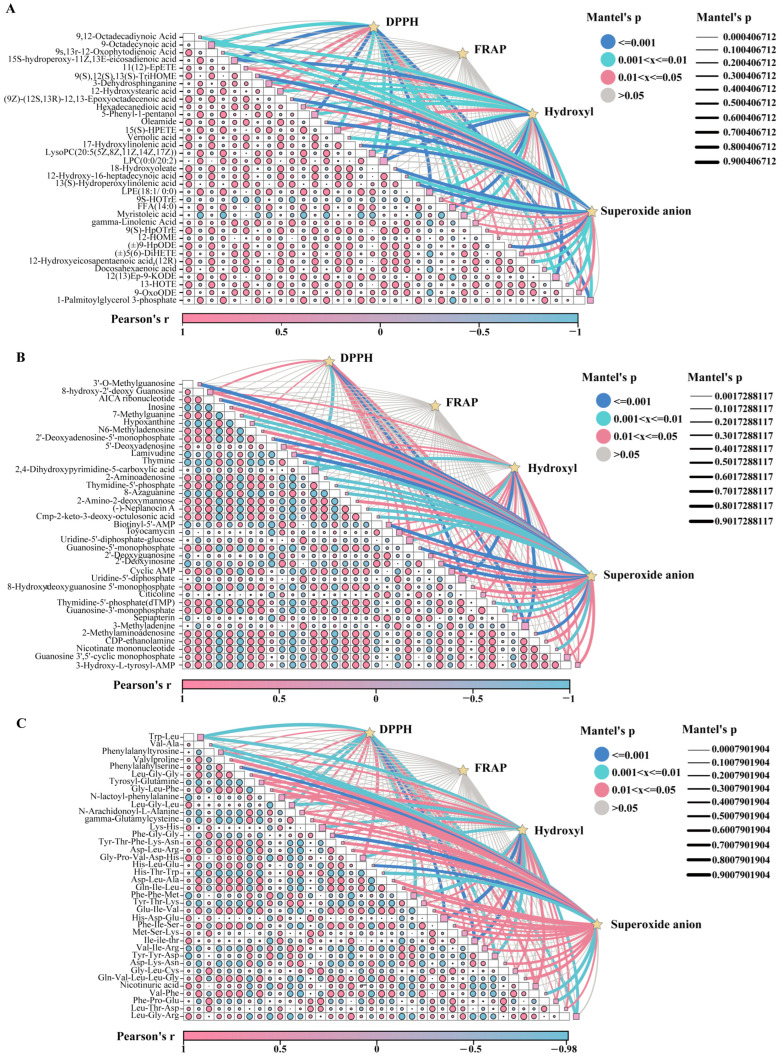
Comparison of antioxidant activity among GL, BL, and RG. (**A**) Interactive Mantel test-based correlation heatmap analysis of DPPH·, FRAP, ^•^OH, O_2_^•−^, and key lipid metabolites. (**B**) Interactive Mantel test-based correlation heatmap analysis of DPPH·, FRAP, ^•^OH, O_2_^•−^, and key nucleotide metabolites. (**C**) Interactive Mantel test-based correlation heatmap analysis of DPPH·, FRAP, ^•^OH, O_2_^•−^, and key amino acid metabolites.

Among the numerous DAMs we identified the top 50 DAMs ranked by variable importance in projection (VIP) values, which were categorized into 11 subgroups for further visual analysis (Figure 7). The results showed that BL displayed significantly elevated expression of amino acid metabolites (h-gamma-Glu-Leu-OH, Glu-Ile-Val, Oxiglutatione, L-Aspartyl-L-phenylalanine, Val-Phe), heterocyclic compound metabolites (3-Hydroxycarbofuran, Cardiospermin, Arborinine, Carnitine C5:0, 3-Methylbutyrylcarnitine), and glycerophospholipid metabolites (LPI (18:2/0:0), LPC (0:0/18:2)). GL exhibited the highest levels of amino acid metabolites (Arginine, His-Ile, Ser-Pro-Asn, His-Trp-Ala, L-Tryptophan, and others), nucleotide metabolites (2′-Deoxyinosine, Hypoxanthine, Toyocamycin), lipid metabolites (Floionolic acid, Oleamide), and alcohol/amine metabolites (2-[(E)-3,7,11,15-tetramethylhexadec-2-enyl]naphthalene-1,4-diol, 2-(Methylthio)ethanol), all of which were significantly upregulated. In contrast, RG showed significantly elevated expression of only four metabolites, namely the amino acid Tyr-Ile-Asn-Glu, the ketone metabolite 4,6-Heneicosanedione, the organic acid metabolite 1-Hydroxyhexanoylglycine, and the alcohol amine metabolite oleoyl ethyl amide. These DAMs are predominantly concentrated in amino acids, nucleotides, and lipids, offering valuable insights for identifying potential biomarkers in *O. sinensis* and its alternative products. Mantel test analysis was conducted to investigate associations between DPPH·, FRAP, ^•^OH, O_2_^•−^ and these key DAMs. Results indicated significant correlations between DPPH·, ^•^OH, O_2_^•−^ and multiple lipid, nucleotide, and amino acid DAMs. Notably, FRAP exhibited no significant correlation with these DAMs (Figure 8A–C). Among lipid DAMs, 3-Dehydrosphinganine, 12-Hydroxystearic acid, Hexadecanedioic acid, and 12-HOME showed a significantly positive correlation with DPPH. Additionally, 11(12)-EpETE, 9S-HOTrE, and (±)5(6)-DiHETE were significantly positively correlated with both ^•^OH and O_2_^•−^. Gamma-linolenic acid and 9-OxoODE were identified as key modulators of DPPH·, ^•^OH, and O_2_^•−^ regulation (Figure 8A). For nucleotide DAMs, 8-hydroxy-2′-deoxy Guanosine, Toyocamycin, Citicoline, Sepiapterin, Guanosine 3′,5′-cyclic monophosphate, and 3-Hydroxy-L-tyrosyl-AMP demonstrated significantly positive correlations with DPPH·, ^•^OH, and O_2_^•−^ (Figure 8B). Most amino acid DAMs, including Tyrosyl-Glutamine, Gly-Leu-Phe, N-Arachidonoyl-L-Alanine, Val-Ile-Arg, and Tyr-Thr-Lys, exhibited a significantly positive correlation with O_2_^•−^ (Figure 8C). These DAMs from *O. sinensis* and its substitutes play a crucial role in the antioxidant activity against ^•^OH, O_2_^•−^, and DPPH·.

## 4. Discussion

There is a close intrinsic association between the medicinal value of *O. sinensis* and its substitutes and their antioxidant activity, with potential differences in the composition of active ingredients among samples from different sources. Based on this, the determination of antioxidant indices of GL, RG, and BL can clarify the material basis underlying their differences in activity, providing crucial phenotypic data support for subsequent exploration of antioxidant mechanisms through metabolomic analysis. In terms of total phenolic content, CAT activity, and DPPH radical scavenging capacity, GL exhibited the highest levels, followed by BL and RG. This phenomenon might be attributed to the unique ecological pressures in the Qinghai–Tibet Plateau, where GL has evolved to accumulate higher levels of phenolic compounds—a class of primary non-enzymatic antioxidants—to counteract oxidative stress induced by extreme environmental factors such as intense ultraviolet radiation and low temperatures [45,46]. Concomitantly, the elevated CAT activity in GL could reflect adaptive upregulation of enzymatic antioxidant systems to synergize with phenolic compounds in neutralizing ROS, thereby enhancing DPPH radical scavenging capacity through the combined action of these molecules, as supported by the significant positive correlations between total phenols, CAT, and DPPH scavenging activity observed in correlation analyses [47]. For SOD activity, POD activity, and O_2_^•−^ scavenging rate, GL again showed the highest levels, with RG ranking second and BL the lowest. This hierarchy may stem from differences in metabolic regulation related to stress adaptation. For instance, wild GL, facing persistent environmental stressors, maintains robust transcriptional and translational machinery for SOD and POD—key enzymes in O_2_^•−^ clearance—to maintain cellular redox homeostasis [48]. RG, while partially retaining the genetic potential for antioxidant enzyme synthesis under control conditions, exhibits reduced induction due to alleviated environmental pressures compared to GL [49]. In contrast, BL, as a fermented product, may undergo metabolic reprogramming during liquid fermentation, leading to downregulated expression or activity of SOD and POD, thereby resulting in weaker O_2_^•−^ scavenging capacity [50]. Notably, BL displayed the highest flavonoid and protein contents, followed by GL and RG. This could be attributed to the distinctive metabolic profiles of fermented products; that is, the liquid deep fermentation process for BL may promote the biosynthesis of flavonoids via enhanced activity of phenylpropanoid pathway enzymes, while the rich nitrogen sources in the fermentation medium might facilitate protein accumulation [51]. In contrast, the slower growth cycle of GL in natural habitats and the nutrient allocation priorities of RG under artificial cultivation may limit the excessive accumulation of these components [52]. It is worth emphasizing that no significant difference was observed in FRAP among the three samples (*p* > 0.05). This finding suggests that the ferric-reducing capacity, which reflects a specific subset of redox-active metabolites, is conserved across GL, RG, and BL, potentially due to the presence of shared redox-active compounds (e.g., certain nucleotides or organic acids) whose levels are maintained within a narrow range regardless of the production mode [53], as indicated by the lack of correlation between FRAP and other antioxidant indices in the Mantel test.

Metabolomics analysis variations in small-molecule metabolites through high-throughput approaches to reveal their associations with physiological changes and identify biomarkers. This technique has been widely applied to analyze metabolite profiles of *O. sinensis* under different conditions. In this study, it was employed in combination with multivariate statistics to conduct metabolite analysis on three sample groups. Metabolites from GL, RG, and BL were classified into 21 superclasses, including amino acids and their derivatives (33.3%), nucleotides and their derivatives, lipids and lipid-like molecules (11.1%), organic acids (10%), alkaloids, and flavonoid compounds. These results are consistent with previous reports on major metabolite categories [54]. In addition, 372 differential metabolites were identified between RG and GL, 176 between BL and GL, and 305 between BL and RG. Studies have shown that the fatty acid content in wild *O. sinensis* is significantly higher than that in artificially cultivated *O. sinensis*, which may be associated with low-temperature stress in the wild growth environment [55]. Among these fatty acids, oleic acid, linoleic acid, and palmitic acid are the major components (accounting for 89.38–94.88% of total fatty acids) and can serve as potential metabolic markers for distinguishing the two [55]. Additional studies have revealed that wild *O. sinensis* is rich in L-pyranose, malic acid, linoleic acid, and oleic acid, while artificially cultivated *O. sinensis* is dominated by sucrose, sorbitol, hydroquinone, nonanoic acid, 1-hydroxy-2-naphthoic acid, thymol-β-D-glucoside, and glycyl-histidine [56]. These compounds with differential contents are all promising characteristic biomarkers for identifying wild *O. sinensis* and artificially cultivated *O. sinensis*. Principal component analysis (PCA) revealed distinct inter-group differences, indicating significant variations in metabolite compositions among GL, BL, and RG. However, GL exhibited notable intra-group variability. KEGG pathway analysis showed that activation of the linoleic acid metabolic pathway was associated with significant downregulation of specific metabolites (e.g., γ-linolenic acid, linoleic acid, lecithin), with differential metabolites across the three groups analyzed in detail. Interestingly, the linoleic acid metabolic pathway was significantly enriched in the GL vs. RG and GL vs. BL comparisons but not in the RG vs. BL comparison. These lipid molecules exhibited a higher relative abundance in GL samples. This phenomenon is likely directly associated with differences in growth environment. GL thrives in extremely high-altitude environments on the Qinghai–Tibet Plateau, where prolonged exposure to stressors such as low temperatures and intense ultraviolet radiation continuously activates the linoleic acid metabolic pathway. By accumulating metabolites like γ-linolenic acid, GL maintains membrane stability and enhances stress resistance and antioxidant capacity, resulting in higher lipid abundance [51]. In contrast, RG and BL are produced in controlled, stable environments. Without the need for high-intensity activation of stress-resistant metabolism, the demand for the linoleic acid pathway is reduced, leading to no significant enrichment in RG and BL [51]. Consistent with our observations, previous studies have reported that wild *O. sinensis* displays elevated relative abundances of lipids and lipoid compounds [57]. It has been reported that flavonoids and terpenoids possess diverse pharmacological activities, including antioxidant, anti-inflammatory, antibacterial, and antitumor effects, as well as cardioprotective properties, blood glucose-lowering capabilities, and preventive effects against respiratory diseases [58,59]. We identified a total of 101 flavonoid metabolites and 149 terpene metabolites. Among these metabolites, 8-glycyrrhizinic acid exerts core biological effects through anti-inflammatory, hepatoprotective, and immunomodulatory activities, effectively alleviating inflammatory responses and protecting hepatocytes [60]. As an anthocyanin compound, delphinidin-3-O-sambudisaccharide delays aging, protects cardiovascular and neural cells by virtue of its potent antioxidant activity [61]. Isogeneity combines antioxidant, anti-inflammatory, and cardiovascular protective properties, while also demonstrating potential antitumor activity [62]. Quercetin 3-xylosyl-(1→6)-glucoside protects cells from oxidative damage through its prominent antioxidant capacity, alongside regulating immune function and metabolic indices [63]. Epicatechin gallate safeguards biological macromolecules via its strong antioxidant characteristics and concurrently exhibits cardiovascular protective, neuroprotective, antibacterial, and antiviral potentials [64,65]. Notably, these metabolites are significantly upregulated in the biosynthetic pathways of RG and BL samples. These findings reveal metabolic discrepancies between wild *O. sinensis* and its substitute products, thereby providing valuable insights into their potential pharmacological characteristics.

Correlation analysis revealed that lipid differential metabolites (12-HOME, 11(12)-EpETE, 9S-HOTrE, (±)5(6)-DiHETE) exhibited significant positive correlations with DPPH radicals, hydroxyl radicals (^•^OH), and superoxide anion free radicals (O_2_^•−^). Most amino acid differential metabolites showed positive correlations with O_2_^•−^. Studies have demonstrated that lipids in *O. sinensis* hold significant value in cardiovascular protection, intervention in inflammatory diseases, adjuvant tumor therapy, and maintenance of skin health [66,67]. Among these lipid molecules, prominent components include γ-linolenic acid, 12-HOME, and 11(12)-EpETE (11,12-EET), alongside other related compounds. Accumulating evidence from research studies has shown that γ-linolenic acid mediates effects such as metabolic regulation, antioxidant activity, anti-inflammatory responses, and immunomodulation via multi-targeted and multi-pathway mechanisms [68,69]. Through untargeted metabolomics analyses, we identified that the significant differences in antioxidant activity between *O. sinensis* and its substitutes are regulated by the linoleic acid metabolic pathway. Additionally, notable variations in lipid-related substances were observed between these samples, leading us to further hypothesize that lipid metabolites may also serve as crucial factors influencing antioxidant capacity. Consequently, lipid metabolomics or targeted lipidomic techniques could be employed to conduct in-depth investigations into the synergistic network of lipid molecules in *O. sinensis*, thereby providing a scientific foundation for the development of novel functional foods and pharmaceuticals [70].

## 5. Conclusions

This study conducted comparative analyses of antioxidant activities and untargeted metabolomics profiles between wild *O. sinensis* and its substitutes. The results indicated that the highest protein and flavonoid contents were observed in fermented Chinese “Bailing” capsules (BL), while they also showed favorable total phenolic content, SOD, POD, and CAT activities, as well as effective scavenging rates against DPPH·, O_2_^•−^, and ^•^OH. Compared with RG and BL, GL displayed significantly stronger antioxidant capacity, confirming its robust antioxidant properties. Untargeted metabolomics analysis revealed that DAMs among wild *O. sinensis*, artificially cultivated *O. sinensis*, and fermented Chinese “Bailing” capsules were primarily concentrated in amino acids and their derivatives, lipid molecules, and nucleotides and their derivatives. Furthermore, KEGG pathway analysis highlighted the synthetic and metabolic pathways associated with these DAMs. Correlation analysis demonstrated that lipid molecules exhibited significant positive correlations with DPPH·, FRAP, ^•^OH, and O_2_^•−^, including key DAMs in the linoleic acid metabolic pathway such as γ-linolenic acid, linoleic acid, and lecithin. DAMs of amino acids and nucleotides showed significant positive correlations with DPPH·, FRAP, ^•^OH, and O_2_^•−^. This study clarified the differences in antioxidant activity characteristics and metabolomics profiles between wild *O. sinensis* and its substitute products, providing a basis for in-depth investigation of key metabolic markers regulating antioxidant function. These findings lay an important foundation for subsequent systematic studies on the antioxidant activity mechanisms and related metabolites of wild *O. sinensis* and its substitutes, while also offering theoretical support for the application of traditional Chinese medicine resources and their substitute raw materials in the development of functional health foods.

## Figures and Tables

**Table 1:** Determination of physicochemical indices for wild *O. sinensis*, artificially cultivated *O. sinensis*, and fermented *O. sinensis* ‘Bailing’ capsules.

Measurement Indicators	Formulas	Note	Absorbance Value
Protein	y = 4.59x + 0.176, R^2^ = 0.9998	BCA method	562 nm
Total polyphenols	total phenol content (mg/g) = 0.356 × (A − 0.0012) ÷ W	A = A_0_ − A_1_, A_0_ is the measured sample, A_1_ is the control sample, and W is the sample quality	760 nm
Flavonoids	flavonoid content (mg/g) = 0.398 × (A − 0.0007) ÷ W	A = A_0_ − A_1_, A_0_ is the measured sample, A_1_ is the blank sample, and W is the sample quality	510 nm
SOD	SOD activit (U/g) = 20 × A ÷ (100 − A) ÷ W	A = (A_0_ − A_1_) ÷ A_0_ × 100%, A_0_ is the control sample, A_1_ is the measured sample, and W is the sample quality	450 nm
POD	POD activit (U/g) = 2000 × A ÷ W	A = A_1_ − A_0_, A_0_ is the absorbance value at one minute, A_1_ is the absorbance value at 2 min, and W is the sample quality	470 nm
DPPH free radical scavenging rate	DPPH free radical scavenging rate (%) = (A_1_ − A_0_) ÷ A_1_ × 100%	A_0_ is the determination sample and A_1_ is the blank sample	515 nm
FRAP antioxidant capacity	y = 2.4832x + 0.0134, R^2^ = 0.9996	X is the concentration of Trolox (μmol/mL)	593 nm
Hydroxyl radical scavenging rate	hydroxyl radical scavenging rate (%) = (A_1_ − A_0_) ÷ (A_1_ − A_2_) × 100%	A_2_, A_1_, and A_0_ are blank tube, control tube and measuring tube, respectively	510 nm
Superoxide anion radical scavenging rate	superoxide anion scavenging rate (%) = (A_1_ − A_0_) ÷ A_1_ × 100%	A_0_ is the determination sample and A_1_ is the blank sample	530 nm

**Table 2:** Gradient separation conditions.

Time (min)	A (%)	B (%)
0.0	95	5
5.0	35	65
6.0	1	99
7.5	1	99
7.6	95	5
1.0	95	5

## Data Availability

The supporting data for the findings of this study are available from the corresponding authors upon reasonable request.

## Supplementary Materials

The following supporting information can be downloaded at: https://www.mdpi.com/article/10.3390/jof11100740/s1, Figure S1: Total Ion Chromatograms (TIC) of QC, GL, BL, and RG under positive mode; Figure S2: Total Ion Chromatograms (TIC) of QC, GL, BL, and RG under positive mode; Table S1: The metabolites present in the samples were detected via untargeted metabolomics.
