# Supplementary figures and images for: Untargeted Metabolomics-Based Characterization of the Metabolic Profile and Antioxidant Activity of Ophiocordyceps sinensis and Its Substitutes
Source: J Fungi (Basel). 2025 Oct 16;11(10):740. doi: 10.3390/jof11100740 (PMC12565446; doi:10.3390/jof11100740)

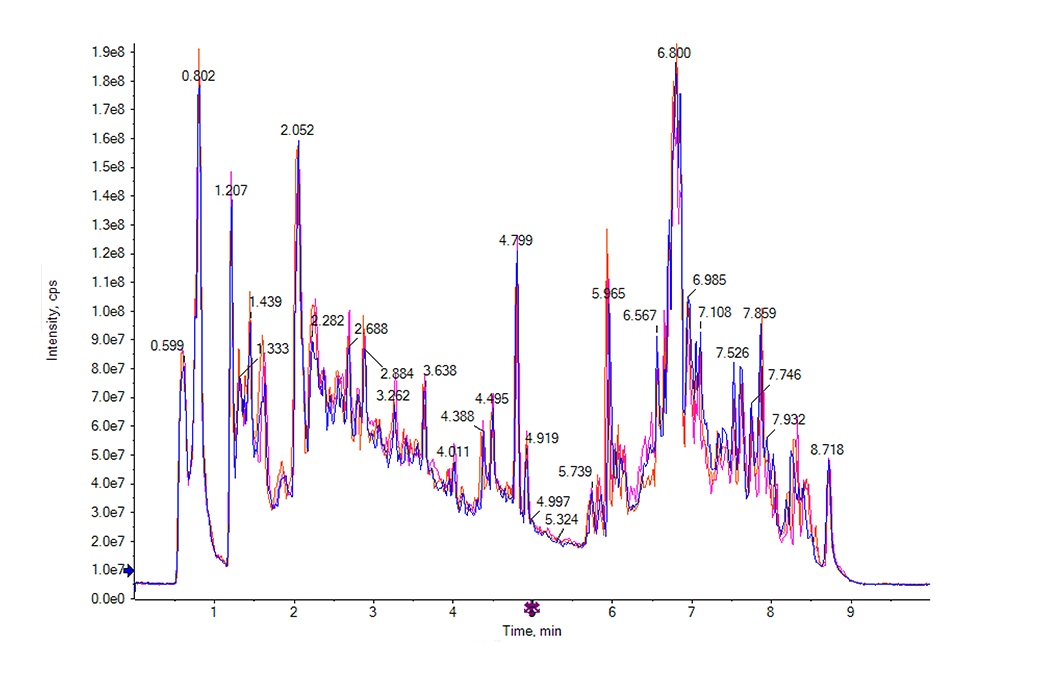

Supplement: Supplementary file 1 [file jof-11-00740-s001.zip › Figure S1.png]

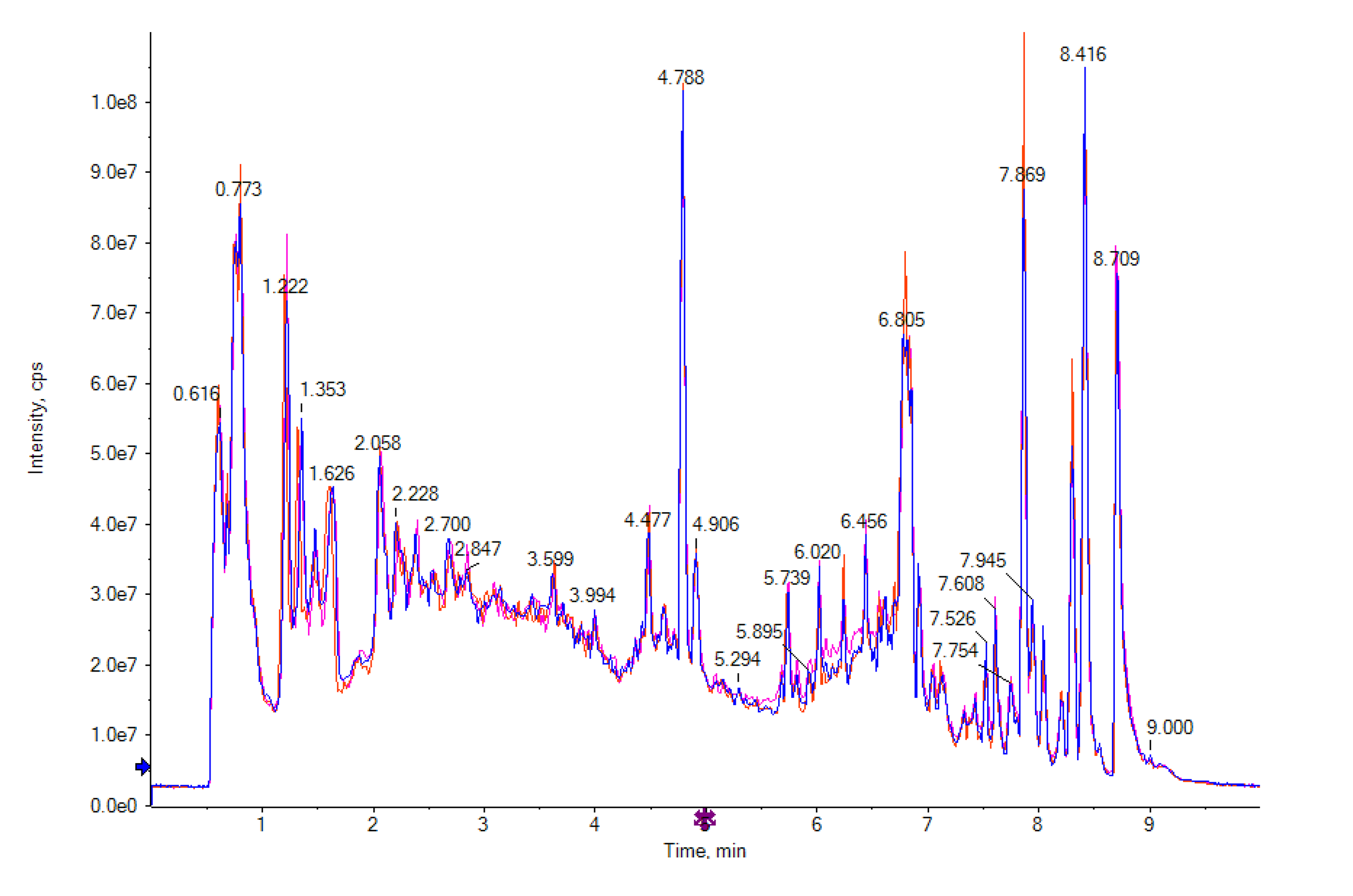

Supplement: Supplementary file 1 [file jof-11-00740-s001.zip › Figure S2.png]
